# Integration of Transcriptomics and Metabolomics Reveals the Responses of Sugar Beet to Continuous Cropping Obstacle

**DOI:** 10.3389/fpls.2021.711333

**Published:** 2021-10-27

**Authors:** Weijuan Huang, Donglei Sun, Ronghua Wang, Yuxing An

**Affiliations:** ^1^Institute of Nanfan & Seed Industry, Guangdong Academy of Sciences, Guangzhou, China; ^2^Shihezi Academy of Agricultural Sciences, Shihezi, China

**Keywords:** continuous cropping obstacle, metabolism, gene expression, RNA-seq, sugar beet

## Abstract

Sugar beet is vulnerable to years of continuous cropping, and allelopathy is one of the important factors leading to continuous cropping disorder. To explore the physiological and molecular mechanisms behind continuous cropping obstacles on sugar beet, this study combined transcriptomics and metabolomics to analyze the effects of different years of continuous cropping on metabolite changes, differential gene expression, and root exudate regulation in sugar beet. We collected sugar beet’s root samples from 1–, 3–, and 5-year continuous cropping systems for metabolome and transcriptome analyses. Our data revealed that T3 and T5 had 50 and 33 metabolites significantly different from T1, respectively. The autotoxic substance salicylaldehyde was found to continuously accumulate in root exudates with increasing years of continuous cropping. Sucrose was highly reduced in T3 (4.05-fold decrease) and T5 (2.01-fold decrease) compared to T1. Respectively, 2,660 and 3,515 differentially expressed genes (DEGs) were significantly regulated in T3 and T5 compared to T1. The Kyoto Encyclopedia of Genes and Genomes (KEGG) enrichment analyses showed that metabolic pathways and biosynthesis of secondary metabolites were perturbed in T3 and T5 vs. T1. Integrated metabolomics analyses identified 73 DEGs involved in enriched metabolic pathways, all of which were the oxidation-reduction process pathways. In conclusion, this study provides evidence that continuous cropping obstacles can change the metabolome and transcriptome of sugar beet, affecting its growth and quality.

## Introduction

Sugar beet (*Beta vulgaris* L. var. *saccharifera*) is one of the two major sugar crops in the world, with a wide cultivation range and considerable economic value. The root tuber is used for sugar production, while the stems and leaves are used for feed and ethanol and biofuel production (Dohm et al., 2014; Finkenstadt, 2014). To obtain high yields with abundant sugar content, reasonable crop rotation and tillage systems have been advocated for in sugar beet production areas (Afshar et al., 2019). However, due to economic interests, limited arable land, and restricted cultivation conditions, continuous cropping is usually required to ensure sugar beet production (Gui and Ji, 2015; Şeker et al., 2017). Long-term continuous cropping can lead to abnormal root development, low resistance to stress, frequent occurrence of diseases and pests, yield quality decline, and even plant death (Jones and Dalal, 2017; Huang C. Z. et al., 2020). Due to factors, such as shortage of cultivated land resources, imperfect cultivation management systems, and production environment constraints (Deihimfard et al., 2019; Holmquist et al., 2021), the problem of continuous cropping obstacles in sugar beet has become increasingly severe and requires urgent solutions.

The mechanisms of continuous cropping obstacle, such as changes in soil physical and chemical properties, accumulation of allelochemicals, and changes in soil microflora, have been attempted to identify in many studies (Qin et al., 2017; Wu et al., 2018; Huang W. et al., 2020). Continuous cropping obstacle is proposed to be the comprehensive effects of plant-soil-microbe interactions (Standing et al., 2007; Inselsbacher et al., 2011). Excessive allelochemicals secreted by roots will stimulate massive population growth of pathogenic microorganisms in the soil, leading to an imbalance of soil microflora (Jin et al., 2019). In our previous study, we detected the dynamic change of the soil rhizosphere microbial community in continuous sugar beet cropping in Xinjiang Uygur Autonomous Region using 16S amplicon sequencing. We found that the relative abundance of *Actinobacteria* decreased sharply with increased continuous cropping years, while populations of *Acidobacteria* and pathogenic fungi, such as *Fusarium* and *Alternaria*, increased significantly (Huang W. et al., 2020). However, the relationship between microbial change and allelochemical accumulation of sugar beet has not been clearly explained.

Allelopathy has also been directly linked to continuous cropping disorder. There are some studies on continuous cropping in rice (Hameed et al., 2019), peanut (Wang et al., 2019), tobacco (Ren et al., 2017), and ginseng (Xiao et al., 2019) which have found that high concentrations of allelopathic substances secreted by roots can produce an autotoxic effect. High levels of toxic substances secreted by sugar beet’s root are known to cause root rot, the blight of sugar beet, brown spot, and other diseases in sugar beets or other crops (Khorassani et al., 2011). However, the main allelopathic substances secreted by sugar beet’s root under continuous cropping and their accumulation characteristics over time have not been fully explored.

**FIGURE 1:**
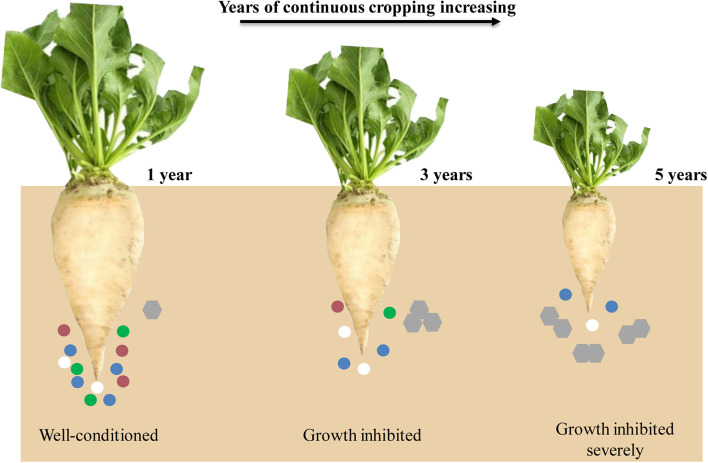
A schematic representation of sugar beet root developmental for all 3 years. Circles and hexagons represent microbes and root exudates, respectively.

In recent years, due to the shortage of cultivated land resources, imperfect cultivation management systems, and restricted production environment in Xinjiang Uygur Autonomous Region, continuous sugar beet cropping has become one of the major agricultural problems. The yield and sugar content of sugar beet have been decreased sharply after years of continuous cropping (Hua et al., 2016); however, the effects of continuous cropping obstacles in sugar beet still lack in-depth investigation. We hypothesized that with years of continuous cropping increasing, the diversity of the rhizosphere microbial community decreased and the root exudates increased, leading to the growth of sugar beet inhibited (Figure 1). Therefore, we have combined transcriptomics and metabolomics to analyze the effects of different continuous cropping years on gene expression and metabolite content of sugar beet, to increase the understanding of the effects of continuous cropping obstacles in this crop.

## Materials and Methods

### Plant Materials and Root Exudates Extraction

Samples of sugar beet (Xintian No. 18 variety) seedlings were collected from 3– and 5-year continuous cropping systems from Shihezi City, Xinjiang Uygur Autonomous Region (86°05′39″N, 44°34′02″E) and 1-year cultivation samples as a control group, with eight replicates in each group (three or more pooled samples per replicate). Sugar beet variety Xintian No. 18 (♀ MS9602A × ♂ SN9807), selected by Shihezi Sugar Beet Research Institute, was a male sterile multigerm diploid variety with resistance to sugar beet rhizomania (Zhang et al., 2009). We sowed the seeds in different plots with 1, 3, and 5 years of continuous cropping separately (three plots for each group, one plot equals 667 m^2^) and harvested the seedlings randomly after growing for 4 weeks. The collected samples were separated into two batches for further process. From the first batch, the roots were washed with tap water, successively rinsed with deionized water three times, cut into small pieces, frozen instantly in liquid nitrogen, and stored at −80°C. From the other batch, the whole seedling was used to obtain root exudates. The sugar beet seedlings were transplanted into a container with sterile deionized water for culturing. The culture process was protected from light and continuously oxygenated. The growth conditions were 25 ± 1°C and a 14-h light/10-h dark cycle. After culturing for 24 h, the culture medium was collected and filtered through a 0.22 μm aqueous phase membrane, and the resulting filtrate contained the root exudates. These were transferred into a 500 ml separation funnel and extracted under a constant volume of ethyl acetate three times. The organic phases were combined and transferred into a rotary evaporation bottle to dry at 50°C. The dried extraction was dissolved in 200-μl isopropanol by vortexing for 2 min and followed by centrifugation at 8,000 × *g* for 15 min at 10°C. The supernatant was subjected to gas chromatography-mass spectrometry (GC-MS) analysis.

### Metabolites Extraction and Metabolomics Analysis

Metabolites of sugar beet root exudates were analyzed by using a non-targeted GC-MS. The samples were derivatized before GC-MS detection using the silanization derivatization method (Mohamed, 2017). The freeze-dried samples were added to 40 μl 20 mg/ml methoxamine pyridine and 10 μl 5% ethyl phenyl acetate/pyridine mixture (ethyl phenyl acetate as internal standard) and placed in an incubator at 60°C for 60 min. Then, 50 μl silanization reagent  *N*,*O*-bis(trimethylsilyl)acetamide (BSTFA) was added and the mixture was incubated at 70°C for 60 min. After natural cooling to room temperature, the mixture was used for GC-MS detection. Data were acquired and processed using Agilent Masshunter version B.04.00 software.

The raw data files generated by GC-MS were processed using the Compound Discoverer 3.0 (CD3.0, Thermo Fisher Scientific, Waltham, MS, United States) to perform peak alignment, peak picking, and quantitation for each metabolite. The main parameters were set as follows: retention time tolerance, 0.2 min; actual mass tolerance, 5 ppm; signal intensity tolerance, 30%; signal/noise ratio, 3; and minimum intensity, 100,000. Peak intensities were normalized to the total spectral intensity. The normalized data were used to predict the molecular formula based on additive ions, molecular ion peaks, and fragment ions. Then peaks were matched with the mzCloud^1^ and ChemSpider^2^ database to obtain accurate qualitative and relative quantitative results.

The metabolites were annotated using the Kyoto Encyclopedia of Genes and Genomes (KEGG) database,^3^ HMDB database,^4^ and LIPID MAPS database.^5^ Principal components analysis (PCA) and partial least squares discriminant analysis (PLS-DA) were performed at metaX (a flexible and comprehensive software for processing metabolomics data) to screen the differences in metabolites between the test group and control group. We applied univariate analysis (*t*-test) to calculate the statistical significance (*P*-value). The metabolites with variable importance in projection (VIP) > 1, *P*-value <0.05, and fold change (FC) ≥ 2 or FC ≤ 0.5 were considered to be differential metabolites. Volcano plots were used to filter metabolites of interest based on Log2(FC) and − log10 (*P*-value) of metabolites.

### Transcriptomic Analysis

Transcriptomic analysis was used to investigate global RNA changes after different years of continuous sugar beet cropping. Total RNA of each sample was extracted using TRIzol Reagent (Invitrogen)/RNeasy Mini Kit (Qiagen, Valencia, CA, United States) and quantified and qualified with an Agilent 2100 Bioanalyzer (Agilent Technologies, Santa Clara, CA, United States), NanoDrop (Thermo Fisher Scientific Inc.), and electrophoresis on 1% agarose gels. One microgram of total RNA with RIN values above 6.5 was used for next-generation sequencing library preparation according to the protocol of the manufacturer. Libraries with different indices were multiplexed and loaded on an Illumina HiSeq instrument according to the instructions of the manufacturer (Illumina, San Diego, CA, United States). The sequences were processed and analyzed by GENEWIZ (South Plainfield, NJ, United States). Differentially expressed genes (DEGs) were selected by using the “DESeq.2” package and analyzed through Student’s *t*-test. Statistical significance was defined as *P* < 0.05 and FC > 10.

### Quantitative Real-Time PCR

The RNA sequence data were verified by Quantitative real-Time PCR (qRT-PCR) analysis. Primer pairs for each target gene were designed and synthesized by GENEWIZ (South Plainfield, NJ, United States). qRT-PCR was conducted using Fast SYBR Green Master Mix (B639271, BBI) on a Light Cycler480 II (Roche, Rotkreuz, Switzerland) instrument. The PCR conditions were as follows: 95°C for 3 min, then 45 cycles of denaturation at 95°C for 7 s, annealing at 60°C for 10 s, and elongation at 72°C for 12 s. GAPDH and β-actin were used to calculate the relative expression level of each target gene through the formula of 2^–ΔΔ^ ^Ct^. Three biological replicates were taken for all qRT-PCR measurements.

### Transcriptomic and Metabolic Integration

MapMan software (Thimm et al., 2004) was used to integrate transcriptomic and metabolic profiles (Moschen et al., 2019). The sugar beet transcriptome (Dohm et al., 2014) was annotated via the Mercator annotation pipeline (Lohse et al., 2014) and used as a library in MapMan. The expression ratio cut off was log_2_ FC higher or lower than 2 (–2) and with a *P*-value lower than 0.05. The resulting data Table was used for MapMan analysis.

### Statistical Analysis

The results were expressed as means ± SE. Significant differences were tested by one-way ANOVA with SPSS 22 (IBM, Armonk, NY, United States). Statistical differences with a *P*-value <0.05 were considered significant.

## Results

### Roots Exudates With Significant Differences During Continuous Cropping

**FIGURE 2:**
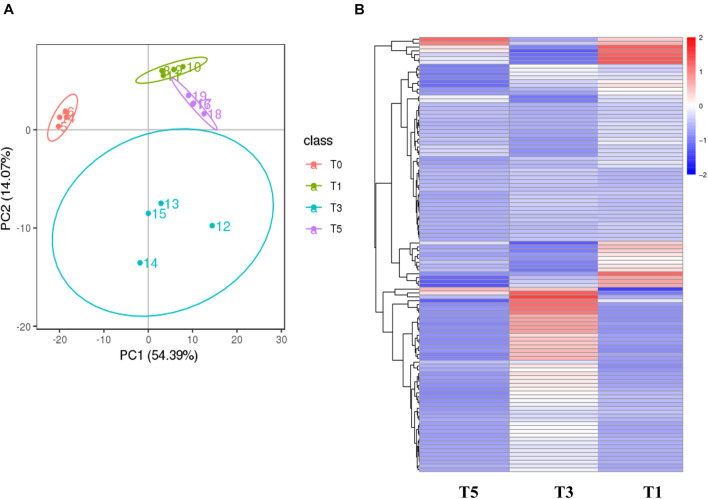
Metabolic profiling results of the monocropping groups (T3 and T5) and control group (T1). **(A)** PLS-DA score plot; **(B)** hierarchical clustering analysis (HCA) for the metabolites based on their z-normalized abundances. T3: 3 years of monocropping; T5: 5 years of monocropping; T1: 1 year of cropping. PLS-DA, partial least squares discriminant analysis.

**FIGURE 3:**
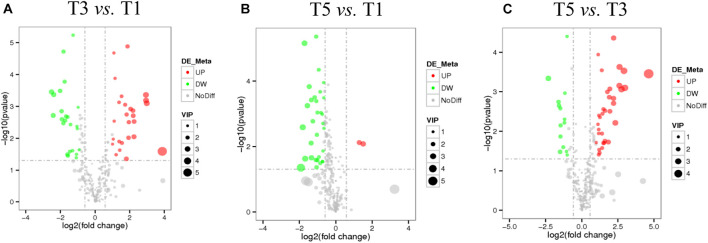
Volcano plots show the important discriminatory metabolites between T3 and T1 **(A)**, between T5 and T1 **(B)**, and between T3 and T5 **(C)**, respectively.

Score plots based on the PLS-DA model showed that the control (T1), 3-year (T3), and 5-year (T5) groups were well separated (Figure 2A). In total, we detected 279 metabolites from sugar beet root exudates through GC-MS analyses. Among these metabolites, 50 (17.9%), 33 (11.8%), and 22 (7.9%) were differentially abundant between T3 and T1, between T5 and T1, and between T5 and T3, respectively (*P* < 0.05, Figure 2B and Supplementary Datas S4–S6). Compared to T1, T3 had 26 metabolites significantly increased and 24 metabolites significantly decreased (*P* < 0.05, VIP > 1, Figure 3A and Table 1). Among them, terephthalic acid (14.74-fold increase), *N*-carbamylglutamate (7.93-fold increase), 1,5-anhydroglucitol (7.71-fold increase), and 5-dihydrocortisone (7.67-fold increase) were produced in greater concentrations in T3; while, 7-alpha-hydroxycholesterol (5.88-fold decrease), pyridoxal phosphate (5.56-fold decrease), sitosterol (5.26-fold decrease), and sucrose (4.17-fold decrease) were greatly reduced in T3 relative to T1 (*P* < 0.05, VIP > 1, Figure 3A and Table 1). Compared to T1, T5 had 31 metabolites significantly decreased and two metabolites significantly increased (*P* < 0.05, VIP > 1, Figure 3B and Table 2). Tetrahydrocorticosterone (3.85-fold decrease), alpha-ketoisocaproic acid (3.57-fold decrease), 1,5-anhydroglucitol (3.23-fold decrease), and palatinitol (3.33-fold decrease) were greatly decreased in T5; while salicylaldehyde (2.83-fold increase) and D-alanyl-D-alanine (2.44-fold increase) were greatly increased in T5 compared to those in T1 (*P* < 0.05, VIP > 1, Figure 3B and Table 2). Compared to T3, T5 had 22 metabolites significantly decreased (*P* < 0.05, VIP > 1, Figure 3C and Table 3). Compared to T5, sucrose (2-fold decrease), L-threose (2.04-fold decrease), glucose (2.08-fold decrease), and isomaltose (2.22-fold decrease) were greatly reduced in T3 (*P* < 0.05, VIP > 1, Figure 3C and Table 3).

**TABLE 1:** Metabolomic analysis of the sugar beet root exudates in T3 compared to T1.

Metabolite	FC	*P* value	VIP	Change
Terephthalic acid	14.74489	0.025861	5.428705	Up
*N*-Carbamylglutamate	7.932363	0.000799	3.026664	Up
5-Dihydrocortisone	7.666312	0.000435	2.990917	Up
1,5-Anhydroglucitol	7.706464	0.00065	2.970821	Up
7-Alpha-Hydroxycholesterol	0.175496	0.00035	2.616602	Down
Sitosterol	0.191518	0.000427	2.486919	Down
5-alpha-Dihydroprogesterone	4.749013	0.008993	2.462498	Up
Pyridoxal phosphate	0.187676	0.001927	2.429155	Down
Fluorene	3.503161	0.04461	2.280455	Up
Fructose	4.777105	0.002983	2.245097	Up
3,4-Dihydroxypyridine	4.605297	0.001964	2.221375	Up
Nornicotine	4.562764	0.00136	2.209108	Up
Indolelactate	4.14295	0.001203	2.118684	Up
Anandamide	3.819407	0.009658	2.115701	Up
Sucrose	0.247109	0.000325	2.09921	Down
Gentiobiose	0.261826	0.001422	2.035457	Down
Glucose	0.264883	0.002524	2.033389	Down
Thymidine	0.330762	0.032472	1.972127	Down
Prostaglandin	3.867495	0.003127	1.959376	Up
Lactamide	0.280514	0.003374	1.951068	Down
Lactitol	3.667307	1.31E-05	1.929916	Up
D-alanyl-D-alanine	3.534306	0.001807	1.883908	Up
Isomaltose	0.282663	1.90E-05	1.87197	Down
Neohesperidin	0.336693	0.035455	1.84487	Down
xylose	0.295488	0.000167	1.822663	Down
21-hydroxypregnenolone	0.302513	0.002766	1.812663	Down
Galactinol	3.336595	0.000888	1.760767	Up
Coprostan-3-one	0.312066	0.002056	1.759421	Down
Dihydroxyacetone	2.932393	0.014074	1.735097	Up
Glucose	0.333662	0.002156	1.665875	Down
Cholestane-3,5,6-triol	0.363735	0.026286	1.665813	Down
*N*-Methyl-DL-alanine	2.889999	0.000481	1.556525	Up
Salicylaldehyde	2.628454	0.012711	1.538297	Up

*FC, fold change; VIP, variable importance in projection. VIP > 1.5 and *P*-value < 0.05 were applied to determine these significantly different metabolites.*

**TABLE 2:** Metabolomic analysis of the sugar beet root exudates in T5 compared to T1.

Metabolite	FC	*P* value	VIP	Change
Tetrahydrocorticosterone	0.264479	0.044503	4.249898	Down
Alpha-ketoisocaproic acid	0.284021	0.002587	2.901862	Down
1,5-Anhydroglucitol	0.311397	0.023519	2.882289	Down
Palatinitol	0.303814	6.98E-06	2.696259	Down
Salicylaldehyde	2.83585	0.008307	2.515365	Up
Glucoheptonic acid	0.338755	0.000565	2.465454	Down
Phenyl beta-D-glucopyranoside	0.395588	0.022133	2.45301	Down
Neohesperidin	0.363351	0.000148	2.267604	Down
D-alanyl-D-alanine	2.446041	0.007545	2.20596	Up
2’-deoxyadenosine	0.390481	0.007822	2.190312	Down
Isoleucine	0.393847	0.000379	2.101082	Down
Tyramine	0.469441	0.025727	1.956187	Down
Cholestane-3,5,6-triol	0.416873	0.001693	1.930549	Down
Cuminic alcohol	0.480998	0.043135	1.83006	down
Mannitol	0.447221	0.000315	1.798286	Down
Sucrose	0.496585	0.026441	1.692724	Down
Phytanic acid	0.473613	4.37E-06	1.68767	Down
Valine	0.476183	0.00041	1.654301	Down
Thymidine	0.486351	0.004391	1.645165	Down
6-phosphogluconic acid	0.49649	0.000917	1.565969	Down

*VIP > 1.5 and *P*-value < 0.05 were applied to determine these significantly different metabolites. VIP, variable importance in projection.*

**TABLE 3:** Metabolomic analysis of the sugar beet root exudates in T5 compared to T3.

Metabolite	FC	*P* value	VIP	Change
Sitosterol	0.200842	0.00046	2.486159	Down
Coprostan-3-one	0.327751	0.001811	1.737619	Down
7-alpha-Hydroxycholesterol	0.335803	0.002257	1.704868	Down
Gluconic lactone	0.353114	0.033191	1.861253	Down
Pyridoxal phosphate	0.35703	0.002638	1.538155	Down
Lactamide	0.359593	0.01358	1.656339	Down
Gentiobiose	0.362087	0.007131	1.62095	Down
Linoleic acid methyl ester	0.411864	0.025269	1.48688	Down
21-hydroxypregnenolone	0.434018	0.006115	1.32491	Down
Xylose	0.437425	0.004981	1.239577	Down
Isomaltose	0.444596	0.001087	1.217599	Down
Glucose	0.479875	0.032572	1.131083	Down
L-Threose	0.492835	3.98E-05	1.075507	Down
Sucrose	0.497616	0.039837	1.01774	Down

*VIP > 1.0 and *P*-value < 0.05 were applied to determine these significantly different metabolites. VIP, variable importance in projection.*

Salicylaldehyde was found to continuously accumulate in root exudates with the increasing years of continuous sugar beet cropping (T3 vs. T1: 2.63-fold increase; T5 vs. T1: 2.84-fold increase). Meanwhile, compared to T1 (Tables 1, 2), sucrose secretion by sugar beet’s root was significantly lower in T3 (4.05-fold decrease) than in T5 (2.01-fold decrease).

### Metabolic Pathway Analysis

Through KEGG pathway enrichment analysis, 89 metabolites of these 279 identified metabolites were mapped onto 19 different KEGG metabolic pathways, including amino acid metabolism, lipid metabolism, carbohydrate metabolism, metabolism of cofactors and vitamins, and biosynthesis of other secondary metabolites. However, the KEGG-enrichment scatterplot showed that there was only one distinguishing pathway, ATP-binding cassette (ABC) transporters, which was significantly enriched in T5 compared to T1 (*P* < 0.05, Supplementary Figure 1). Three distinct metabolites were annotated to this KEGG pathway, such as sucrose, mannitol, and phosphate. T3 vs. T1 and T5 vs. T3 had no significantly distinguishing KEGG pathway.

### Transcriptomics Profiling of Sugar Beet in Response to Continuous Cropping Obstacle

**FIGURE 4:**
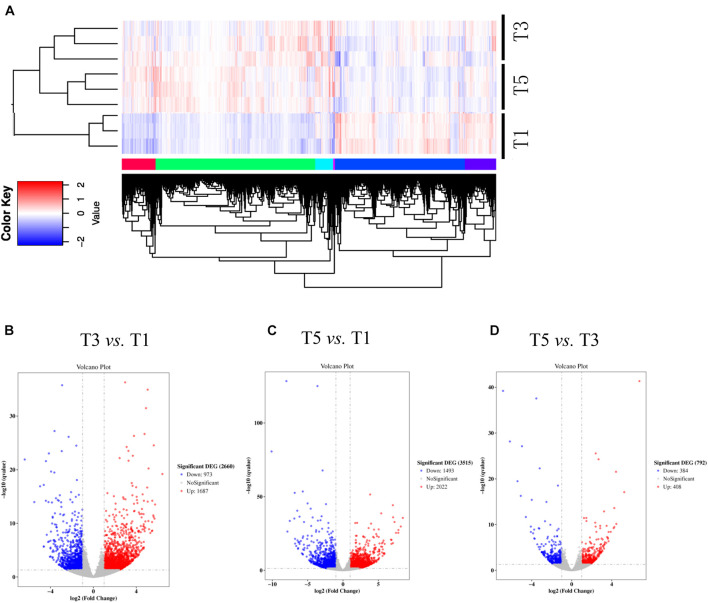
Numbers of differentially expressed genes (DEGs) in sugar beet’s root in response to **(A)** 3 years of continuous cropping (T3), **(B)** 5 years of continuous cropping (T5), and **(C)** the comparative DEGs between T3 and T5. **(D)** Heatmap shows the profiling of DEGs between T1, T3, and T5.

Table 4 displays a total of 42,715,073, 42,178,983, and 41,263,831 raw reads that were obtained from T1, T3, and T5, respectively. After removing low-quality sequences, 42,665,359, 42,133,136, and 41,214,037 clean reads were retained and used for assembly. The RNA-Seq results indicated that the total numbers of genes expressed were distinct between different years of continuous sugar beet cropping (Figure 4A). Specifically, 1,687 genes were significantly upregulated, and 973 genes were significantly downregulated in the T3 group compared to the T1 group (Figure 4B and Supplementary Data S1). Total 2,022 genes were significantly upregulated and 1,493 genes were significantly downregulated in the T5 group compared to the T1 group (Figure 4C and Supplementary Data S2), and 408 genes were significantly upregulated, and 384 genes were downregulated in the T5 group compared to the T3 group (Figure 4D and Supplementary Data S3). Nine DEGs (Supplementary Table 1) randomly selected for qRT-PCR verification showed similar expression patterns compared with the RNA-seq assay (Supplementary Figure 2), ensuring the reliability of the transcriptome sequencing data.

**TABLE 4:** Summary statistics of RNA sequencing results.

Group	Total raw reads	Total clean reads	Multiple mapped	Uniquely mapped	Q20 (%)	GC (%)
T1	42,715,073	42,665,359	1590077	36,257,139	97.52	44.06
T3	42,178,983	42,133,136	1714520	35,188,196	97.42	43.43
T5	41,263,831	41,214,037	1614049	35,218,029	97.50	43.86

*Multiple mapped: statistics of the number of sequences with multiple alignment positions on the reference sequence; Uniquely mapped: statistics of the number of sequences with unique alignment positions on the reference sequence.*

### Gene Oncology and Kyoto Encyclopedia of Genes and Genomes Enrichment Analyses

To illustrate the association between DEGs and metabolic pathways, we conducted two pathway-based analyses using the Gene Oncology (GO) and KEGG databases. GO pathway analysis focused on the significantly affected genes in the treatment groups (T3 and T5) compared with those in the control group (T1). A summary of the overrepresented GO terms in the T3 and T5 groups is displayed in Table 5. Significantly altered DEGs in T3 compared to T1 were functionally annotated in several major metabolic pathways related to oxidoreductase activity, oxidation-reduction process, and integral component of membrane (Supplementary Figure 3A). Compared to T1, the altered DEGs in T5 were mainly related to the oxidation-reduction process, transmembrane transport, and metabolic process (Supplementary Figure 3B). In addition, T5 was over enriched in the plant-type cell wall pathway compared to T3 (Supplementary Figure 3C).

**TABLE 5:** Top overrepresented GO groups for the comparison of groups T1 vs. T3, T1 vs. T5, and T3 vs. T5.

Comparison between groups	GO terms
T1 vs T3	Integral component of membrane(GO:0016021), cell redox homeostasis(GO:0045454), oxidation-reduction process(GO:0055114), protein disulfide oxidoreductase activity(GO:0015035), electron carrier activity(GO:0009055), isoprenoid biosynthetic process(GO:0008299), hydrolase activity, hydrolyzing O-glycosyl compounds(GO:0004553), starch synthase activity(GO:0009011), glucan biosynthetic process(GO:0009250), glycerol ether metabolic process(GO:0006662)
T1 vs T5	Oxidation-reduction process(GO:0055114), transmembrane transport(GO:0055085), metabolic process(GO:0008152), oxidoreductase activity(GO:0016491), integral component of membrane(GO:0016021)
T3 vs T5	Plant-type cell wall(GO:0009505)

*GO, gene oncology.*

**FIGURE 5:**
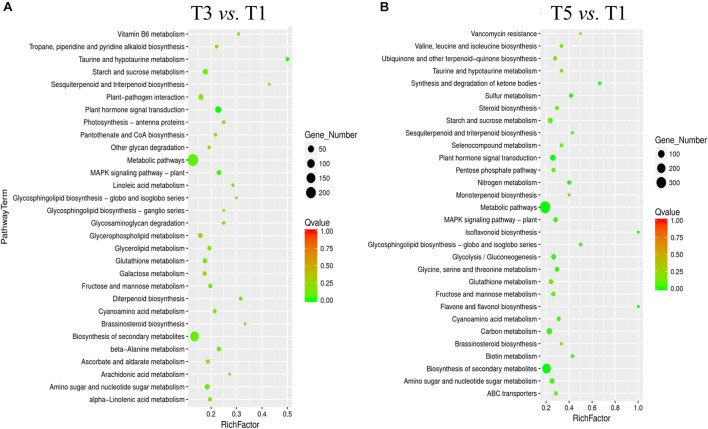
KEGG pathway enrichment for DEGs in **(A)** T3 vs. T1, and **(B)** T5 vs. T1. DEGs, differentially expressed genes.

The Kyoto Encyclopedia of Genes and Genomes enrichment analysis provided an overview of alterations to metabolism in the biological processes in response to different years of continuous cropping. KEGG enrichment analyses showed that metabolic pathways (T3: 242 DEGs and T5: 360 DEGs) and biosynthesis of secondary metabolites (T3: 140 DEGs and T5: 211 DEGs) were perturbed in the T3 and T5 groups vs. the T1 group, suggesting the close relationship between these DEGs and metabolites (Figures 5A,B). In addition, the carbohydrate metabolism pathways were significantly enriched with continuous croppings, such as starch and sucrose metabolism (T3: 24 DEGs and T5: 32 DEGs) and amino sugar and nucleotide sugar metabolism (T3: 22 DEGs and T5: 30 DEGs).

### Joint Analysis of Metabolomics and Transcriptomics Data

**FIGURE 6:**
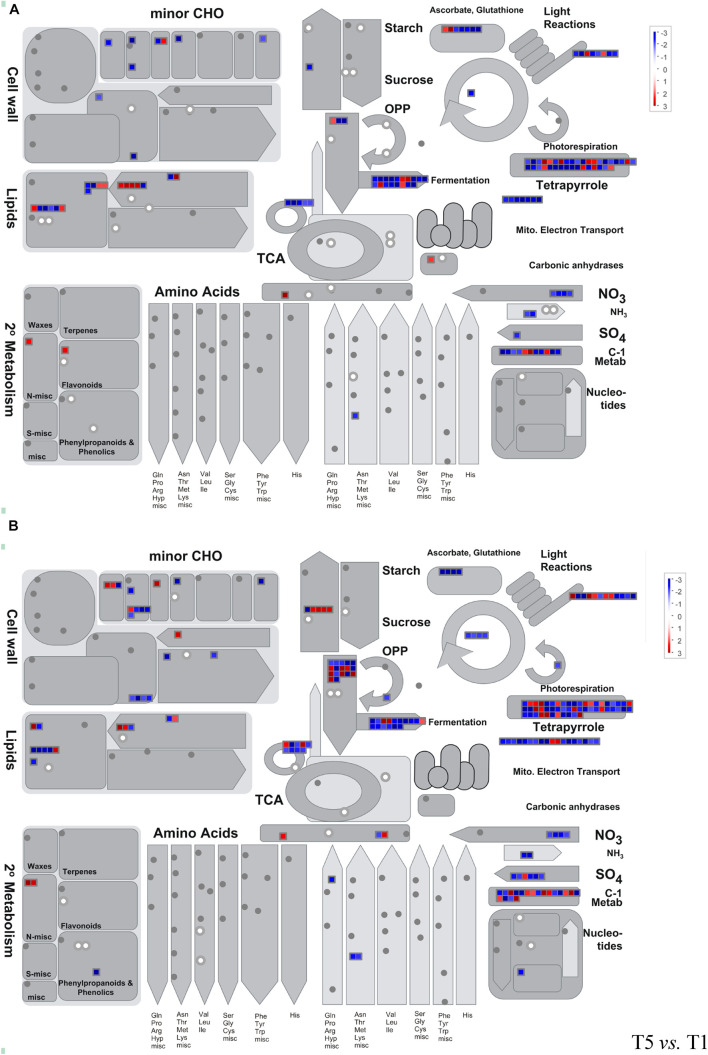
Metabolism overview using MapMan. **(A)** T3 vs. T1; **(B)** T5 vs. T1. Genes and metabolites are represented by squares and circles, respectively. Color intensity corresponds to the expression ratio at the logarithmic scale (log2). Red: upregulated, blue: downregulated.

A pathway-based approach combining metabolomics and transcriptomics analysis was conducted to better understand the relationship between dysregulated genes and metabolites. Here, we employed MapMan analysis to compare the different biological processes in T3 vs. T1 and T5 vs. T1. In Figure 6, we found downregulation of genes and metabolites associated with photosynthesis and photorespiration, cell wall modification, tetrapyrrole, nitrogen and sulfur metabolism, and fermentation process in T3 and T5 compared to T1. Whereas, genes related to secondary metabolites, sugar, and starch degradation showed upregulation in both T3 and T5 vs. T1.

In addition, 73 DEGs (43 upregulated and 30 downregulated, false discovery rate (FDR) < 0.05) in total were identified as enriched metabolic pathways using metabolomics, and GO annotation analysis was performed on these DEGs. All were involved in the oxidation-reduction process pathway (GO: 0055114; Supplementary Figure 4). A total of eight KEGG pathways were obtained repeatedly from both metabolomics and transcriptomics data-based pathway analysis (Supplementary Figure 5 and Tables 2, 3). Among the eight KEGG pathways, only one pathway of starch and sucrose metabolism showed significantly reduced expression in T3 and T5 compared to T1.

## Discussion

Continuous cropping obstacle is a complex problem that has plagued agricultural production for a long time. The mechanisms behind its formation and its means of prevention have always been researched hotspots. As reported, continuous cropping obstacle has a great impact on the growth characteristics of sugar beet, such as abnormal root development, low resistance to stress, frequent occurrence of diseases and pests, yield quality decline, and even plant death (Nie et al., 2008; Bainard et al., 2013; Yigezu et al., 2019). In this study, we combined transcriptome and metabolome analysis to reveal the effects of continuous cropping obstacles on the gene expression and metabolite change in sugar beet. Through studying sugar beet at different years of continuous cropping, we explored the internal mechanisms driving changes in the metabolite components and inferred the effects of continuous cropping obstacles on the growth and development of sugar beet.

We identified the metabolites excreted from sugar beet’s root, including terephthalic acid, salicylaldehyde, and neohesperidin in the exudates of sugar beet, was significantly increased in the monocropping groups (T3 and T5) compared to the control group (T1). For example, salicylaldehyde was found to continuously accumulate in root exudates with increased continuous cropping (Tables 1–3). Especially, the terephthalic acid content in the plots of T3 was nearly 14 times higher than for T1, suggesting it is the most likely autotoxic substance related to continuous cropping obstacle in sugar beets. The reasons leading to a high accumulation of allelochemicals in the sugar beet’s root could be 1) a massive reproduction of pathogenic microorganisms in the soil including *Fusarium* spp. and *Alternaria* spp. as we have found previously (Huang W. et al., 2020); and 2) change of soil nutritional status after long-term monocropping (Wang et al., 2012; Haichar et al., 2014). Through long-term evolution, plants have to adjust the composition and amount of root exudates to respond positively to changes in the surrounding environment (Hu et al., 2018; Korenblum et al., 2020). It is widely accepted that the content of autotoxic substances secreted by plant roots would be accumulated with the increasing years of continuous cropping, which could produce an autotoxic effect, leading to the occurrence of continuous cropping obstacles (Huang et al., 2013; Cheng and Cheng, 2015; Li et al., 2016). In sugar beet, Li et al. (2016) found that the root exudates 2,6-dit-butyl p-cresol and phthalic acid have inhibitory effects on the growth and development of soybean. This study showed that salicylaldehyde and D-alanyl-D-alanine were also highly accumulated in T5 vs. T3. Sucrose content was lower in T3 and in T5 compared to T1 (Table 3). Further research studies are needed to verify whether any of these differentially expressed secondary metabolites (Tables 1–3) directly cause autotoxic effects on sugar beet.

Furthermore, root exudates from the plants with continuous cropping can stimulate the massive breeding of soil pathogenic microorganisms, leading to the imbalance of soil microflora (Hu et al., 2018). For example, succinic acid, p-hydroxybenzoic acid, p-coumaric acid, and glutamate in cucumber root exudates were found to be able to reduce the diversity index and change the structure of the soil bacterial community (Jin et al., 2019). In tobacco, members of the rhizosphere microbial community, such as *Bacillus subtilis* and *Bacillus amylolyticus*, increased significantly after the induction of plants with jasmonic acid (Zhalnina et al., 2018). In our previous study, we found that continuous sugar beet cropping resulted in a significant decline in the diversity of soil microbial community and continuous structural changes in soil bacterial and fungal populations from T1 to T5 (Huang W. et al., 2020). However, the effect of allelochemicals was secreted by continuous sugar beet cropping on the rhizosphere microbial community structure and the related regulatory mechanisms require further investigation.

The transcriptomics data revealed that 2,660 and 3,515 DEGs were significantly alerted in T3 and T5, respectively, compared to T1. The analysis of transcriptomic data revealed that these DEGs were mainly distributed in the metabolic and biosynthesis of secondary metabolites on the KEGG pathways (Figure 5), suggesting that the significantly upregulated DEGs could be involved in the synthesis of root exudates. More studies are required to understand how these different genes are involved in regulating the biosynthesis of root exudation. From the result of KEGG-enrichment analysis, we found that there was only one distinguishing pathway, ABC transporters (such as, gene-LOC104884804, gene-LOC104904870, and gene-LOC104888312; Supplementary Table 3), which was significantly enriched in T5 compared to T1 (*P* < 0.05, Supplementary Figure 1). ABC transporters are involved in the transport of secondary metabolites (Yazaki, 2006; Dahuja et al., 2021). Under abiotic stress, the ABC transporters transport various secondary metabolites, such as terpenoids, quinones, alkaloids, and polyphenols (Theodoulou, 2000). Meanwhile, the biosynthesis and distribution of secondary metabolites inside the plant are highly regulated and their accumulation in different organs depends on the ABC transporters (Yazaki, 2006). The secondary metabolites detected in the root exudates of T5, including salicylaldehyde, were also revealed much higher than T1 (Table 2), indicating that these enriched ABC transporter pathways could be highly relevant to the accumulated secondary metabolites in T5. In addition, the flavonoid biosynthesis pathway has only one DEG (gene-LOC104886776) significantly enriched in T3 *vs.* T1, while seven DEGs significantly enriched in T5 vs. T1, such as LOC104907876 encoding flavonoid 3′-monooxygenase and LOC104908900 encoding flavonoid 3′,5′-methyltransferase-like, indicating the flavonoids-related metabolites may become increased and diverse with the effects of long-term monocropping. However, genes encoding the ABC transporter of secondary metabolites and flavonoid biosynthesis in plant species are abundant, which still requires more researches to find the target genes and check the genes expression.

The GO terms suggest that the most significantly enriched terms were the oxidation-reduction process (GO: 0055114), which included 37 genes upregulated and 17 genes downregulated in T3 vs. T1 (Supplementary Data S7), and 43 genes upregulated and 30 genes downregulated in T5 vs. T1 (Supplementary Data S8). For example, genes (LOC104895143, LOC104895152) annotated to alcohol dehydrogenase were downregulated, while gene (LOC104898398) annotated to monodehydroascorbate reductase (MDHAR) was upregulated. MDHAR is an important enzyme in the ascorbic acid-glutathione cycle (AsA-GSH), which catalyzes the reduction of MDHA to AsA and plays an important role in the redox metabolism of AsA (Gallie, 2013). The 2.3-fold and 1.8-fold upregulation of MDHAR-related genes in T5 and T3 observed in this study suggested that increased years of continuous cropping may lead to the increased susceptibility of sugar beet to the soil environment stress (Tan et al., 2021). In the plant-pathogen interaction pathway, both T3 and T5 had over 20 DEGs enriched compared to T1, which revealed that those DEGs could have responses to pathogens that increased in the soil after years of continuous cropping (Huang W. et al., 2020; Lau et al., 2020).

By combining transcriptome and metabolome analyses, it is found that continuous sugar beet cropping correlates with distinctive changes in metabolic process gene expression and metabolite variations in sugar beet’s root, with metabolites and genes in the same pathway always dysregulated together, and thus, we used an integrative analysis to present a comprehensive overview between the differential metabolites and DEGs. The metabolic pathways and biosynthesis of secondary metabolites pathways were the most DEG pathways with continuous cropping and could be highly relevant to the synthesis of allelopathic substances in sugar beet’s root in response to continuous cropping obstacles.

## Data Availability Statement

The original contributions presented in the study are publicly available. This data can be found here: National Center for Biotechnology Information (NCBI) BioProject database under accession number PRJNA730685.

## Author Contributions

WH and YA designed this work and drafted and revised the manuscript. DS and RW conducted the field survey and collected the samples. WH conducted the laboratory assays. All authors read and approved the final version of the manuscript.

## Conflict of Interest

The authors declare that the research was conducted in the absence of any commercial or financial relationships that could be construed as a potential conflict of interest.

## Publisher’s Note

All claims expressed in this article are solely those of the authors and do not necessarily represent those of their affiliated organizations, or those of the publisher, the editors and the reviewers. Any product that may be evaluated in this article, or claim that may be made by its manufacturer, is not guaranteed or endorsed by the publisher.

## Footnotes

1
https://www.mzcloud.org/

2
http://www.chemspider.com/

3
http://www.genome.jp/kegg/

4
http://www.hmdb.ca/

5
http://www.lipidmaps.org/
