# Supplementary material for: Integration of Transcriptomics and Metabolomics Reveals the Responses of Sugar Beet to Continuous Cropping Obstacle
Source: Front Plant Sci. 2021 Oct 27;12:711333. doi: 10.3389/fpls.2021.711333 (PMC8578061; doi:10.3389/fpls.2021.711333)
Supplement: Supplementary file 1 [file Data_Sheet_1.zip › Supplementary Figures 1-5.PDF]

Supplementary Figures:

Supplementary Figure 1 Scatter plot of the top KEGG pathway enrichment for differential metabolites in the T5 and T1 groups.

Supplementary Figure 2 RT-qPCR verification. 1, NADPH dehydrogenase; 2, Cytochrome C; 3, NAD(P)H-nitrite reductase; 4, Glycosyl transferases ; 5, Glutamine Synthetase; 6, Glutamate synthase; 7, Glutamate dehydrogenase; 8, Superoxide dismutase; 9, β-actin.

Supplementary Figure 3 GO pathway enrichment for (**A**) differential metabolites in T3 versus T1, (**B**) differential metabolites in T5 versus T1, and (**C**) differential metabolites in T5 versus T3.

Supplementary Figure 4 GO pathway in the biological process in the T5 group versus the T1 group. Oxidation reduction pathway (GO:0055114) is significantly enriched in T5 versus in T1, which could be relevant to the stress of continuous cropping obstacles.

Supplementary Figure 5 Venn plot of metabolic pathways enriched by metabolomics and transcriptomics. (A) T3 *vs*. T1, (B) T5 *vs*. T1.

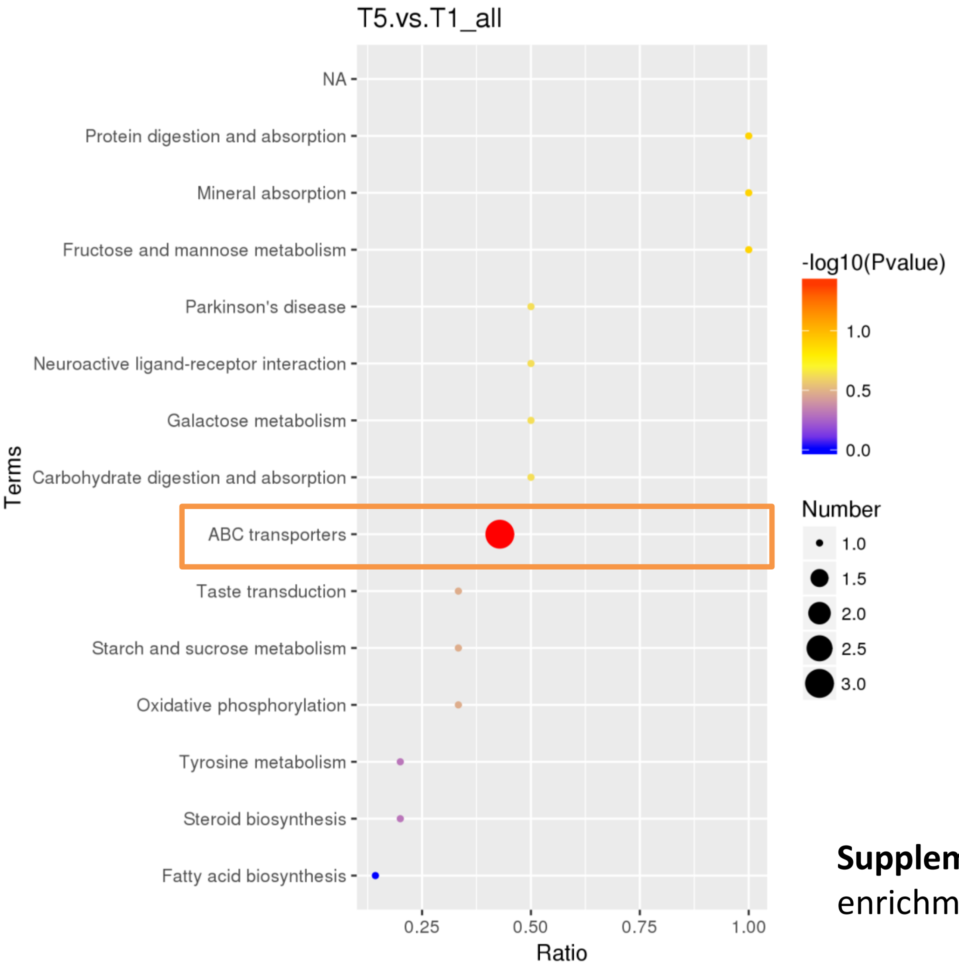

Supplementary Figure 1 Scatter plot of the top KEGG pathway enrichment for differential metabolites in the T5 and T1 groups.

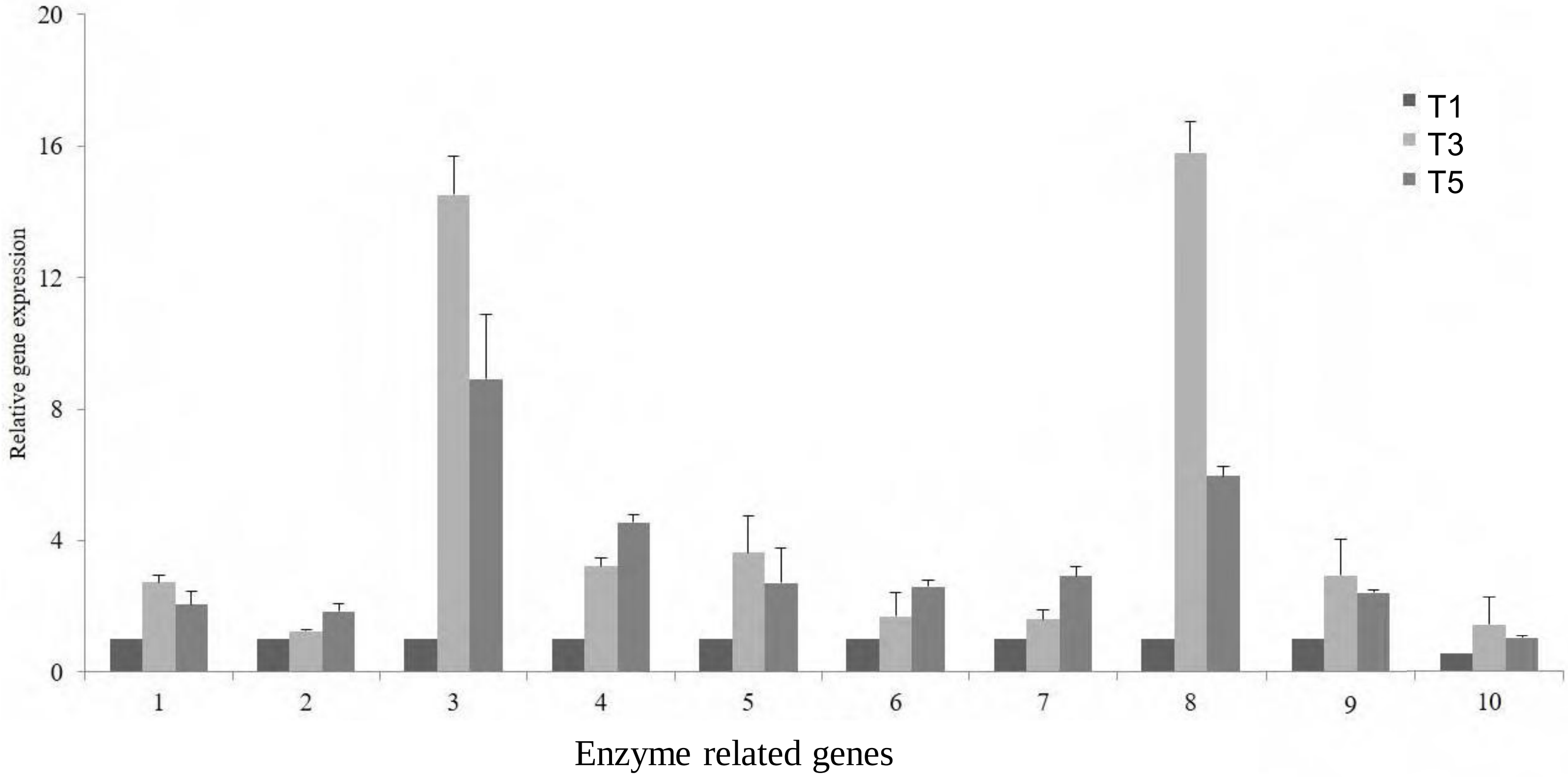

Supplementary Figure 2 RT-qPCR verification.

1, NADPH dehydrogenase; 2, Cytochrome C; 3, NAD(P)H-nitrite reductase; 4, Glycosyl transferases ; 5, Glutamine Synthetase; 6, Glutamate synthase; 7, Glutamate dehydrogenase; 8, Superoxide dismutase; 9, β-actin; 10, GAPDH.

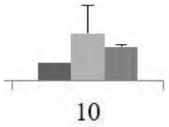
10

A

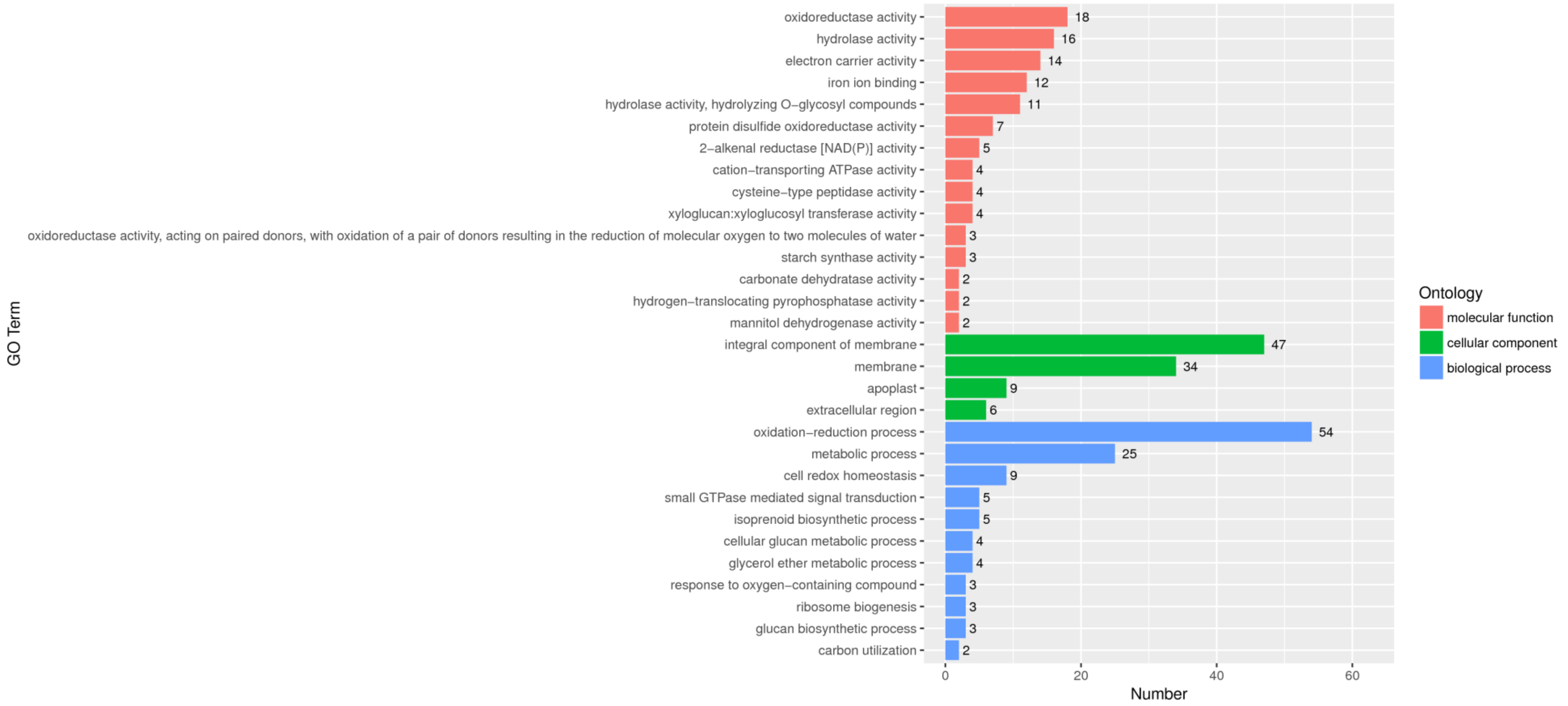

Supplementary Figure 3 GO pathway enrichment
for (**A**) differential metabolites in T3 versus T1, (**B**) differential metabolites in T5 versus T1, and (**C**) differential metabolites in T5 versus T3.

B

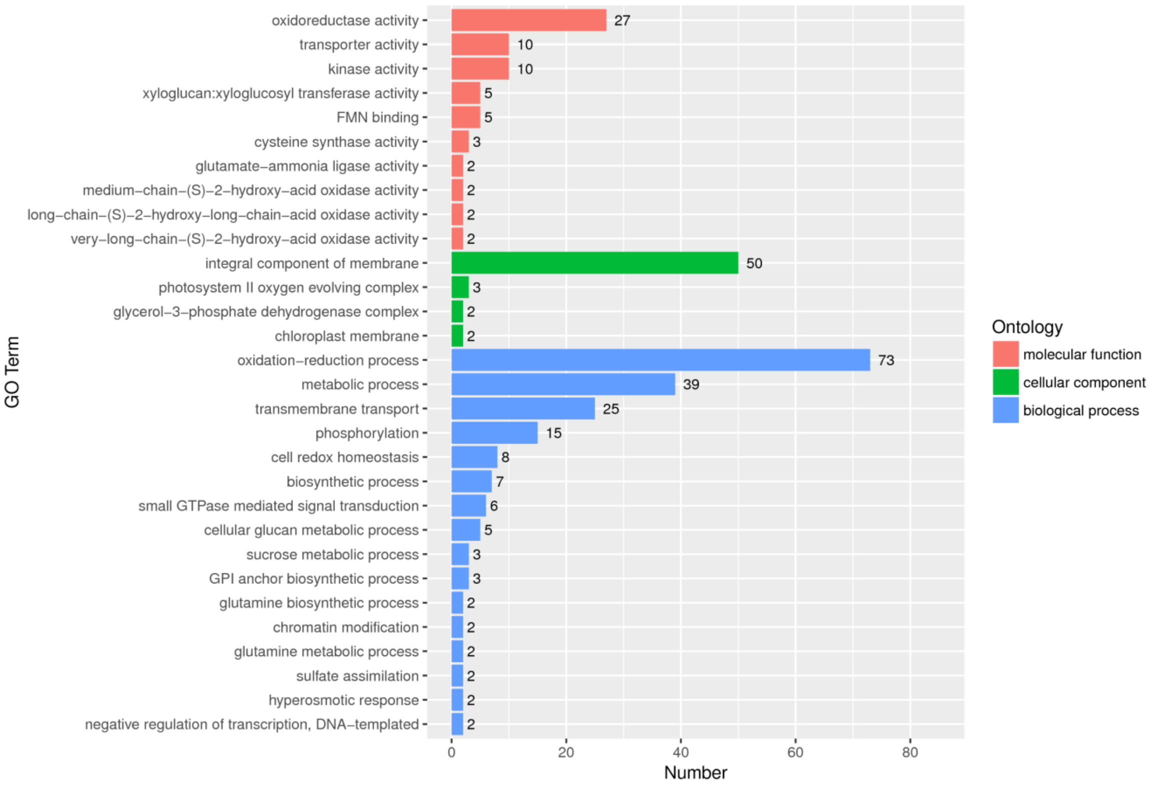

oxidoreductase activity
transporter activity
kinase activity
xyloglucan:xyloglucosyl transferase activity
FMN binding
cysteine synthase activity
glutamate–ammonia ligase activity
medium–chain–(S)–2–hydroxy–acid oxidase activity
long–chain–(S)–2–hydroxy–long–chain–acid oxidase activity
very–long–chain–(S)–2–hydroxy–acid oxidase activity
integral component of membrane
photosystem II oxygen evolving complex
glycerol–3–phosphate dehydrogenase complex
chloroplast membrane
oxidation–reduction process
metabolic process
transmembrane transport
phosphorylation
cell redox homeostasis
biosynthetic process
small GTPase mediated signal transduction
cellular glucan metabolic process
sucrose metabolic process
GPI anchor biosynthetic process
glutamine biosynthetic process
chromatin modification
glutamine metabolic process
sulfate assimilation
hyperosmotic response
negative regulation of transcription, DNA–templated
27
10
10
5
5
3
2
2
2
2
50
3
2
2
73
39
25
15
8
7
6
5
3
3
2
2
2
2
2
2
GO Term
0
20
40
60
80
Number
Ontology
molecular function
cellular component
biological process

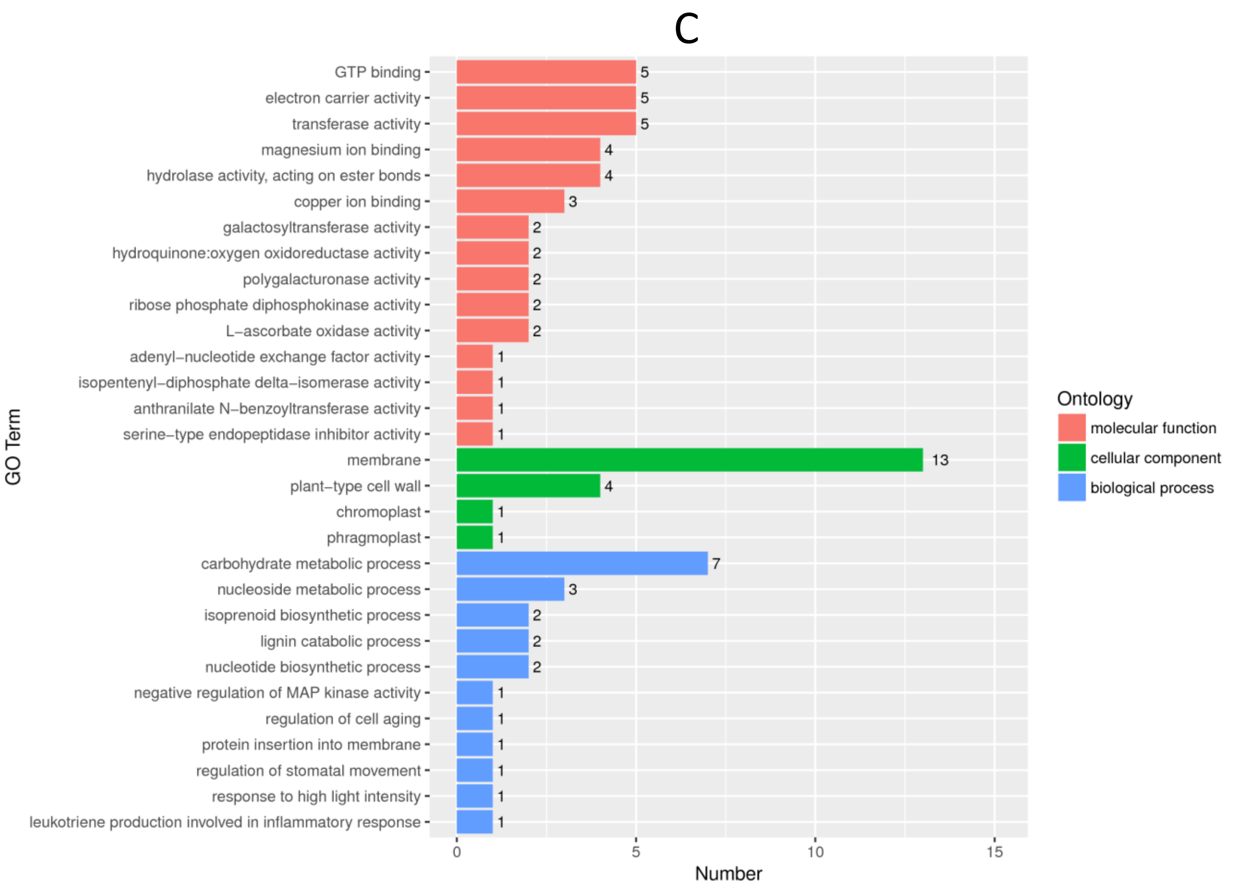
C
GTP binding
electron carrier activity
transferase activity
magnesium ion binding
hydrolase activity, acting on ester bonds
copper ion binding
galactosyltransferase activity
hydroquinone:oxygen oxidoreductase activity
polygalacturonase activity
ribose phosphate diphosphokinase activity
L–ascorbate oxidase activity
adenyl–nucleotide exchange factor activity
isopentenyl–diphosphate delta–isomerase activity
anthranilate N–benzoyltransferase activity
serine–type endopeptidase inhibitor activity
membrane
plant–type cell wall
chromoplast
phragmoplast
carbohydrate metabolic process
nucleoside metabolic process
isoprenoid biosynthetic process
lignin catabolic process
nucleotide biosynthetic process
negative regulation of MAP kinase activity
regulation of cell aging
protein insertion into membrane
regulation of stomatal movement
response to high light intensity
leukotriene production involved in inflammatory response
5
5
5
4
4
3
2
2
2
2
2
1
1
1
1
13
4
1
1
7
3
2
2
2
1
1
1
1
1
1
GO Term
0
5
10
15
Number
Ontology
molecular function
cellular component
biological process

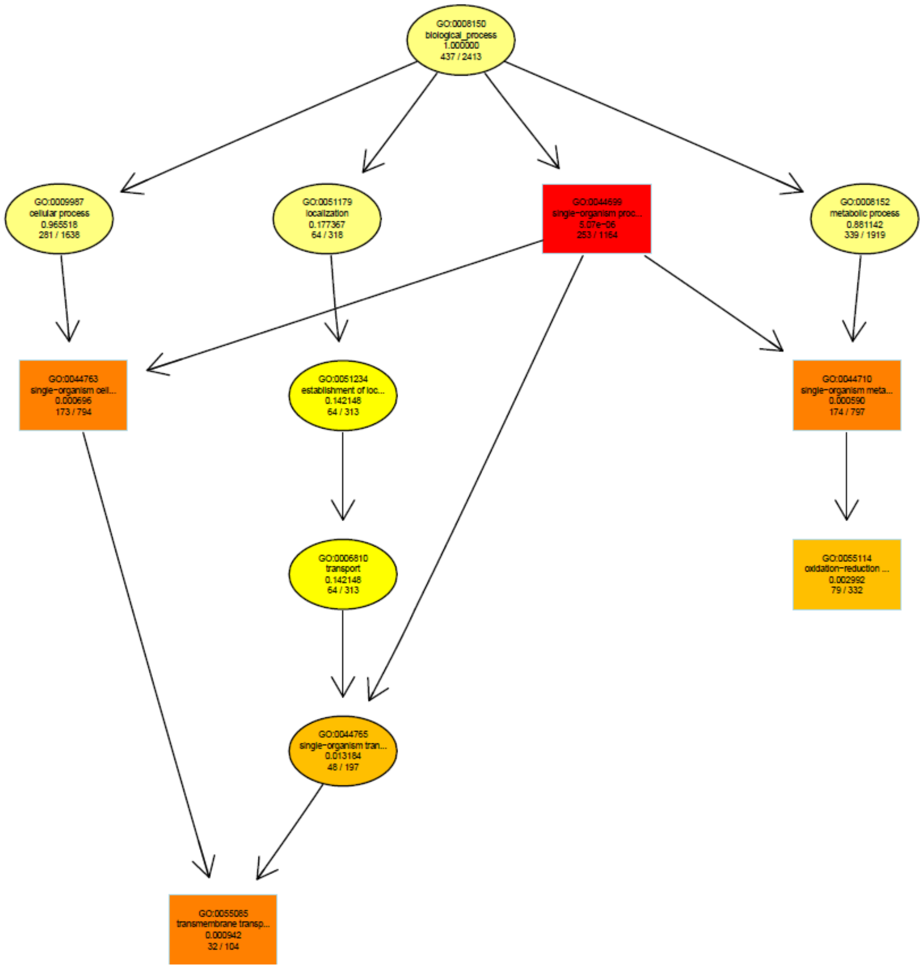

Supplementary Figure 4 GO pathway in the biological process in the T5 group versus the T1 group. Oxidation reduction pathway (GO:0055114) is significantly enriched in T5 versus in T1, which could be relevant to the stress of continuous cropping obstacles.

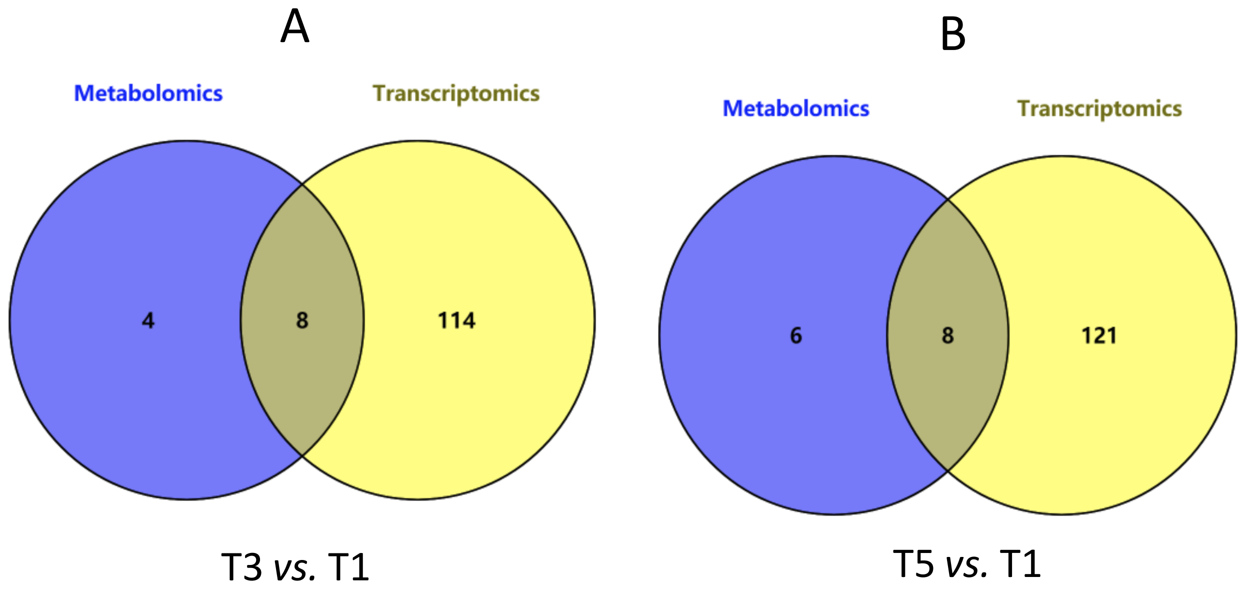

Supplementary Figure 5 Venn plot of metabolic pathways enriched by metabolomics and transcriptomics. (A) T3 *vs.* T1, (B) T5 *vs.* T1.
