# Supplementary material for: Integration of Transcriptomics and Metabolomics Reveals the Responses of Sugar Beet to Continuous Cropping Obstacle
Source: Front Plant Sci. 2021 Oct 27;12:711333. doi: 10.3389/fpls.2021.711333 (PMC8578061; doi:10.3389/fpls.2021.711333)
Supplement: Supplementary file 1 [file Data_Sheet_1.zip › Supplementary Tables 1-3.DOCX]

**Supplementary Table 1 Specific primers of RT-qPCR amplification**

| Gene ID | Gene name | Primer(5'-3') |
| --- | --- | --- |
|  |  |  |
| gene-LOC104904096 | NADPH dehydrogenase | F-TCCTGGCTCTGCTTCAACAACC  R-GACGACGGTGACCTTCTTCTCC |
| gene-LOC104904819 | Cytochrome C | F-ATGGCACACTGAAATCGCATCG  R-TGGCTCCTCCTTCTTTGGTTCC |
| gene-LOC104887841 | NAD(P)H-nitrite reductase | F-GCCGCTGGATTTGATGCTGATT  R-TGCACCAACACCGACAACAAC |
| gene-LOC109133531 | Glycosyl transferases | F-GCCTCACCGACTCAGACACCTT  R-GATTTGGCGGCGGAGGAAGT |
| gene-LOC104883503 | Glutamine synthetase | F-GCTATTGGAGCGGACAAATCGT  R-CCACTGACCAGGCATCACTTCT |
| gene-LOC104888263 | Glutamate synthase | F-AGAGCAAGTGGGTGGCAAAGG  R-GGGCAACCGCATTTCTTCCAAT |
| gene-LOC104888710 | Glutamate dehydrogenase | F-ACAACGAGATTCGCCGATTCTG  R-CGTCCGCCAACACCAATATCAC |
| gene-LOC104887645 | Superoxide dismutase | F-TCACCACCAGAAGCACCATCAA  R-GGACAACCTTGGAAGCATCCCT |
|  | β-actin | F-GGGGATGAAGCACAGTCCAA  R-GCCGTGGTTGTGAAGGAGTA |
|  | GAPDH | F-GTGGTTTCCAACGCATCCTG  R-GCTCTCCAGTCCTTACCTCCCT |

**Supplementary Table 2 Eight common KEGG pathways between T3 and T1.**

| Pathway ID | Pathway | DEGs with pathway annotation (527) | P value | Gene list |
| --- | --- | --- | --- | --- |
| ko00100 | Steroid biosynthesis | 6(1.14%) | 0.084815 | gene-LOC104905231,gene-LOC104889439,gene-LOC104901642,  gene-LOC104904268,gene-LOC104901952,gene-LOC104906256, |
| ko00052 | Galactose metabolism | 11(2.09%) | 0.049624 | gene-LOC104895650,gene-LOC104887530,gene-LOC104903729,  gene-LOC104883129,gene-LOC104883130,gene-LOC104886631,  gene-LOC104887650,gene-LOC104885917,gene-LOC104899812,  gene-LOC104889159,gene-LOC104892324, |
| ko00561 | Glycerolipid metabolism | 11(2.09%) | 0.024619 | gene-LOC104889439,gene-LOC104904546,gene-LOC104897405,  gene-LOC104904633,gene-LOC104894797,gene-LOC104909051,  gene-LOC104896551,gene-LOC104886631,gene-LOC104887650,  gene-LOC104893966,gene-LOC104890532, |
| ko01200 | Carbon metabolism | 21(3.98%) | 8.74E-01 | gene-LOC104904633,gene-LOC104903729,gene-LOC104883129,  gene-LOC104891831,gene-LOC104897526,gene-LOC104898495,  gene-LOC104889100,gene-LOC104889468,gene-LOC104883130,  gene-LOC104892681,gene-LOC104884255,gene-LOC109133905,  gene-LOC104905987,gene-LOC104897452,gene-LOC104885917,  gene-LOC104897815,gene-LOC104906385,gene-LOC104898563,  gene-LOC104883596,gene-LOC104904770,gene-LOC104907006, |
| ko02010 | ABC transporters | 6(1.14%) | 9.61E-02 | gene-LOC104884804,gene-LOC104887441,gene-LOC104884018,  gene-LOC104904870,gene-LOC104902345,gene-LOC104888312, |
| ko00190 | Oxidative phosphorylation | 8(1.52%) | 8.62E-01 | gene-LOC104904547,gene-LOC104901923,gene-LOC104886535,  gene-LOC104902389,gene-LOC104898772,gene-LOC104890297,  gene-LOC104888955,gene-LOC104904901, |
| ko00500 | Starch and sucrose metabolism | 24(4.55%) | 9.03E-03 | gene-LOC104906215,gene-LOC104909120,gene-LOC104897773,  gene-LOC104892160,gene-LOC104883129,gene-LOC104883130,  gene-LOC104892576,gene-LOC104889921,gene-LOC104892603,  gene-LOC104891859,gene-LOC104889614,gene-LOC104883661,  gene-LOC104895698,gene-LOC104908344,gene-LOC104891604,  gene-LOC104900375,gene-LOC104891740,gene-LOC104888235,  gene-LOC104889559,gene-LOC104892054,gene-LOC104909043,  gene-LOC104898951,gene-LOC104892392,gene-LOC104890172, |
| ko01100 | Metabolic pathways | 242(45.92%) | 3.94E-03 | gene-LOC104901461,gene-LOC104886776,gene-LOC104906215,gene-LOC104893391,gene-LOC104892088,gene-LOC104905231,gene-LOC104901937,gene-LOC104907238,gene-LOC104908975,gene-LOC104889439,gene-LOC104901642,gene-LOC104887530,gene-LOC104897369,gene-LOC104904546,gene-LOC104906403,gene-LOC104905946,gene-LOC104897405,gene-LOC104906437,gene-LOC104895114,gene-LOC104890023,gene-LOC104909120,gene-LOC104905703,gene-LOC104904633,gene-LOC104897773,gene-LOC104904425,gene-LOC104884391,gene-LOC104902120,gene-LOC104902048,gene-LOC104903729,gene-LOC104903407,gene-LOC104890234,gene-LOC104895358,gene-LOC104904943,gene-LOC104909093,gene-LOC104892160,gene-LOC104888140,gene-LOC104883129,gene-LOC104905695,gene-LOC104891831,gene-LOC104902364,gene-LOC104892095,gene-LOC104897526,gene-LOC104890160,gene-LOC104898495,gene-LOC104892045,gene-LOC104888263,gene-LOC104900946,gene-LOC104908689,gene-LOC104884038,gene-LOC104895476,gene-LOC104890101,gene-LOC104895292,gene-LOC104888130,gene-LOC104887706,gene-LOC104908933,gene-LOC104892526,gene-LOC104889100,gene-LOC104891300,gene-LOC104894037,gene-LOC104889468,gene-LOC104893978,gene-LOC104906088,gene-LOC104904096,gene-LOC104888246,gene-LOC104889892,gene-LOC104896272,gene-LOC104900715,gene-LOC104907820,gene-LOC104892360,gene-LOC104906606,gene-LOC104905363,gene-LOC104883130,gene-LOC104904268,gene-LOC104906150,gene-LOC104898916,gene-LOC104906111,gene-LOC104903665,gene-LOC104900250,gene-LOC104883006,gene-LOC104887991,gene-LOC104897360,gene-LOC104892576,gene-LOC104894011,gene-LOC104897615,gene-LOC104892334,gene-LOC104906170,gene-LOC104898398,gene-LOC104885747,gene-LOC104889907,gene-LOC104894797,gene-LOC104905398,gene-LOC104898866,gene-LOC104895152,gene-LOC104901992,gene-LOC104905390,gene-LOC104888149,gene-LOC104892681,gene-LOC104895955,gene-LOC104899089,gene-LOC104882838,gene-LOC104887764,gene-LOC104888376,gene-LOC104898523,gene-LOC104889921,gene-LOC104884389,gene-LOC104888401,gene-LOC104892603,gene-LOC104890995,gene-LOC104889056,gene-LOC104898566,gene-LOC104899357,gene-LOC104902718,gene-LOC104883993,gene-LOC104884255,gene-LOC104909051,gene-LOC104906695,gene-LOC104905095,gene-LOC109133718,gene-LOC104887508,gene-LOC104902346,gene-LOC104889614,gene-LOC104896551,gene-LOC104892989,gene-LOC104894457,gene-LOC104897633,gene-LOC104883661,gene-LOC104894995,gene-LOC104896721,gene-LOC104903428,gene-LOC109136359,gene-LOC104883634,gene-LOC104906405,gene-LOC104895698,gene-LOC104906016,gene-LOC104883144,gene-LOC109133905,gene-LOC104891609,gene-LOC104906288,gene-LOC104890297,gene-LOC104908017,gene-LOC104884478,gene-LOC104892003,gene-LOC104905987,gene-LOC104891028,gene-LOC104901563,gene-LOC104904801,gene-LOC104899685,gene-LOC104901952,gene-LOC104903391,gene-LOC104888955,gene-LOC104891604,gene-LOC104899336,gene-LOC104903433,gene-LOC104900375,gene-LOC104893663,gene-LOC104897452,gene-LOC104890411,gene-LOC104899916,gene-LOC104889186,gene-LOC104899125,gene-LOC104889885,gene-LOC104892649,gene-LOC104902735,gene-LOC104897872,gene-LOC104893405,gene-LOC104908025,gene-LOC104891587,gene-LOC104895712,gene-LOC104901785,gene-LOC104893204,gene-LOC104907864,gene-LOC104893966,gene-LOC104901913,gene-LOC104900981,gene-LOC104900052,gene-LOC104889932,gene-LOC104907355,gene-LOC104885917,gene-LOC104891740,gene-LOC104891675,gene-LOC104884969,gene-LOC104895532,gene-LOC104897815,gene-LOC104891737,gene-LOC104897378,gene-LOC104905734,gene-LOC104895418,gene-LOC104888235,gene-LOC104883981,gene-LOC104890532,gene-LOC104897009,gene-LOC104898351,gene-LOC104902326,gene-LOC104906385,gene-LOC104889559,gene-LOC104899812,gene-LOC104892054,gene-LOC104888697,gene-LOC104901861,gene-LOC104889159,gene-LOC104898563,gene-LOC104892324,gene-LOC104889314,gene-LOC104883596,gene-LOC104884500,gene-LOC104906256,gene-LOC104897618,gene-LOC104900274,gene-LOC104904880,gene-LOC104904770,gene-LOC104898583,gene-LOC104909043,gene-LOC104902204,gene-LOC104898951,gene-LOC104907707,gene-LOC104898206,gene-LOC104899677,gene-LOC104887587,gene-LOC104897235,gene-LOC104892392,gene-LOC104894597,gene-LOC104896148,gene-LOC104906946,gene-LOC104898910,gene-LOC104895531,gene-LOC104887297,gene-LOC104904013,gene-LOC104896499,gene-LOC104901768,gene-LOC104886641,gene-LOC104897902,gene-LOC104888141,gene-LOC104907006,gene-LOC104907596,gene-LOC104901553,gene-LOC104890172,gene-LOC104905183,gene-LOC104907232,gene-LOC104902399,gene-LOC104905264,gene-LOC104895516,gene-LOC104887771, |

**Supplementary Table 3 Eight common KEGG pathways between T5 and T1.**

| Pathway ID | Pathway | DEGs with pathway annotation (527) | P value | Gene list |
| --- | --- | --- | --- | --- |
| ko02010 | ABC transporters | 10(1.33%) | 1.94E-02 | gene-LOC104892207,gene-LOC104884804,gene-LOC104904870,  gene-LOC104902295,gene-LOC104905624,gene-LOC104906200,  gene-LOC104898642,gene-LOC104888312,gene-LOC109134573,  gene-LOC104888315 |
| ko00051 | Fructose and mannose metabolism | 16(2.12%) | 1.51E-02 | gene-LOC104883129,gene-LOC104883130,gene-LOC104899125,  gene-LOC104901368,gene-LOC104903729,gene-LOC104905363,  gene-LOC104885917,gene-LOC104892005,gene-LOC104900934,  gene-LOC104906170,gene-LOC104902340,gene-LOC104901753,  gene-LOC104892392,gene-LOC104902429,gene-LOC104906399,  gene-LOC104904724, |
| ko00052 | Galactose metabolism | 13(1.73%) | 1.35E-01 | gene-LOC104883129,gene-LOC104883130,gene-LOC104903729,  gene-LOC104885917,gene-LOC104888351,gene-LOC104895650,  gene-LOC104886632,gene-LOC104907700,gene-LOC104887650,  gene-LOC104902340,gene-LOC104894535,gene-LOC104896820,  gene-LOC104906396, |
| ko00190 | Oxidative phosphorylation | 11(1.46%) | 9.39E-01 | gene-LOC104901923,gene-LOC104898772,gene-LOC104898366,  gene-LOC104892886,gene-LOC104890297,gene-LOC104886535,  gene-LOC104887564,gene-LOC104892651,gene-LOC104891690,  gene-LOC109133834,gene-LOC104899661, |
| ko00500 | Starch and sucrose metabolism | 32(4.25%) | 8.68E-03 | gene-LOC104882830,gene-LOC104905748,gene-LOC104906215,  gene-LOC104883129,gene-LOC104883130,gene-LOC104891294,  gene-LOC104882831,gene-LOC104889614,gene-LOC104892603,  gene-LOC104892576,gene-LOC104897773,gene-LOC104894247,  gene-LOC104891740,gene-LOC104888351,gene-LOC104890172,  gene-LOC104884072,gene-LOC104883661,gene-LOC104900440,  gene-LOC104889559,gene-LOC104907700,gene-LOC104889921,  gene-SBSS1,gene-LOC104908344,gene-LOC104896820,  gene-LOC104891604,gene-LOC104906396,gene-LOC104892392,  gene-LOC104892160,gene-LOC104900375,gene-LOC104900089,  gene-LOC104894964,gene-LOC104906399, |
| ko00100 | Steroid biosynthesis | 10(1.33%) | 1.55E-02 | gene-LOC104905231,gene-LOC104901642,gene-LOC104902610,  gene-LOC104893142,gene-LOC104907279,gene-LOC104904269,  gene-LOC104901952,gene-LOC104901954,gene-LOC104892421,  gene-LOC104894986, |
| ko00350 | Tyrosine metabolism | 13(1.73%) | 5.36E-02 | gene-LOC104895152,gene-LOC104892088,gene-LOC104892566,  gene-LOC104902228,gene-LOC104902495,gene-LOC104908658,  gene-LOC104884255,gene-LOC104902718,gene-LOC109135688,  gene-LOC104884038,gene-LOC104891903,gene-LOC104892124,  gene-LOC104895143, |
| ko00061 | Fatty acid biosynthesis | 8(1.06%) | 8.91E-02 | gene-LOC104901389,gene-LOC104902742,gene-LOC104890177,  gene-LOC104897631,gene-LOC104908919,gene-LOC104889901  ,gene-LOC104892559,gene-LOC104888726, |
